# Patient-reported adherence to physical exercises of patients with ankylosing spondylitis

**DOI:** 10.1007/s10067-022-06189-w

**Published:** 2022-05-03

**Authors:** Sai Ma, Liang Zhang, Siliang Man, Tao Bian, Hongchao Li, Weiyi Li, Zhuyi Ma, Da He

**Affiliations:** 1 Department of Spine Surgery, Beijing Jishuitan Hospital, Fourth Clinical College of Peking University, No. 31 Xinjiekou East Street, Xicheng District, Beijing, 100035 China; 2 Department of Orthopedic Surgery, Beijing Jishuitan Hospital, Fourth Clinical College of Peking University, No. 31 Xinjiekou East Street, Xicheng District, Beijing, 100035 China; 3 Department of Rheumatology, Beijing Jishuitan Hospital, Fourth Clinical College of Peking University, No. 31 Xinjiekou East Street, Xicheng District, Beijing, 100035 China; 4 Department of Physical Therapy and Rehabilitation, Beijing Jishuitan Hospital, Fourth Clinical College of Peking University, No. 31 Xinjiekou East Street, Xicheng District, Beijing, 100035 China

**Keywords:** Adherence, Ankylosing spondylitis, Logistic regression model, Rehabilitation

## Abstract

**Introduction:**

Studies on adherence to exercise therapy of patients with ankylosing spondylitis (AS) are rare, and the criteria for adherence to exercise are inconsistent. This study aimed to quantify patient-reported adherence to exercise therapy of Chinese outpatients with AS and investigate the factors related to poor adherence.

**Methods:**

The subjects’ sociodemographic, disease-related, radiographic, and laboratory parameters were collected. Patients’ adherence to exercise therapy was assessed using the Exercise Attitude Questionnaire (EAQ) with a 4-point Likert scale. All cases were grouped as good adherence and poor adherence using a cutoff score of 60, according to a previous study. Univariate analysis was conducted to assess the intergroup differences. Then, we built a multivariate logistic regression model to identify possible significant factors related to poor adherence to exercise therapy.

**Results:**

A total of 185 outpatients completed the questionnaire. The mean EAQ score was 49.4 (IQR, 40.7–59.3) and 146 patients (78.9%) were considered to have poor adherence, and 39 patients (21.1%) were considered to have good adherence. The rates of current nonsteroidal anti-inflammatory drugs (NSAIDs), conventional synthetic disease-modifying antirheumatic drugs (csDMARDs), and tumor necrosis factor-α inhibitor (TNF-i) use were significantly higher in the poor adherence group (*p*=0.001, *p*=0.027, *p*=0.018, respectively). Our multivariate logistic regression model revealed that the only significant associated factor was current use of NSAIDs (OR=3.517; *p*=0.016; 95% CI, 1.259–9.827).

**Conclusions:**

Outpatients with AS had an unacceptable level of adherence to exercise therapy, and current use of NSAIDs was a significantly associated factor.

**Table:** 

**Key Points** *• Outpatients with AS had an unacceptable level of adherence to exercise therapy.* *• Current use of NSAIDs exerted a negative impact on patients’ adherence to exercise therapy.*

## Introduction

Ankylosing spondylitis (AS) is a disease prototype of a heterogeneous group of inflammatory arthritis known as seronegative spondyloarthropathies [1, 2]. AS mainly affects the spine and sacroiliac joints, causing characteristic inflammatory back pain. If untreated, this may progress to severe damage to the spine, sacroiliac joint, and peripheral joints with functional impairment, reduction of physiological range of motion (ROM), deformity, disability, and compromised psychological status and quality of life [3–5].

Adherence, defined by the World Health Organization (WHO) as “the extent to which a person’s behavior-taking medications, following a diet, and/or executing lifestyle changes corresponds with agreed recommendations from the health care provider,” is an extremely important determinant in the good control of AS. High rates of poor adherence to prescribed medications by patients with AS have been reported in some previous studies [6–9] and the consequences of this include increased disease activity and disability, increased number of disease flares, and increased healthcare costs [10, 11].

Although the treatment of AS has been revolutionized with the advent of biological therapies, rehabilitation is still a promising nonpharmacological therapy in AS, which usually include aerobic exercise, respiratory kinesiotherapy, strengthening, stretching, and balance and gait training. In AS, the main goals of exercise focus on pain control, prevention and delay of stiffness, improvement of function and gait, and correction of deformity in combination with pharmacological therapy [12, 13]. However, reports on adherence to physical exercises in patients with AS are relatively rare [7, 8, 14], and the criteria for adherence to exercises have been inconsistent.

The Exercise Attitude Questionnaire (EAQ) was introduced previously for the evaluation of adherence to exercise therapy [15] and its reliability and validity have been confirmed [8, 15]. To the best of our knowledge, this was the first study to assess adherence to exercise therapy of Chinese outpatients with AS by EAQ. The aims of the study were to quantify patient-reported adherence to exercise therapy and to investigate the possible factors related to poor adherence.

## Materials and methods

### Patient enrollment

This cross-sectional study included 232 consecutive outpatients who fulfilled the modified New York criteria [16] for AS at Beijing Jishuitan Hospital from February 2019 to September 2021. All patients gave their informed consent prior to inclusion in the study. The exclusion criteria were as follows: (1) age ≥ 50 years or ≤ 18 years; (2) any diagnosis of psychiatric disorders and currently on psychiatric treatments, and cognitive dysfunction; and (3) incomplete questionnaires.

### Patient demographics and clinical characteristics

The participants’ sociodemographic factors included body mass index (BMI), sex, age at outpatient visit, family history, education level (primary school, junior middle school, senior middle school or professional education, and university/graduate education), working status (employed, unemployed, or retired), and smoking habits (current or past).

The disease-related characteristics included age at onset of AS, duration of AS, diagnosis delay, visual analog scale (VAS) of global back pain, extra-articular manifestations (EAMs) (current or past) including uveitis, psoriasis, and inflammatory bowel disease (IBD), and medication status. Diagnosis delay was defined as the interval between a patient’s first spondyloarthritis symptoms and the correct diagnosis of AS. The use of medications including nonsteroidal anti-inflammatory drugs (NSAIDs), conventional synthetic disease-modifying antirheumatic drugs (csDMARDs), and tumor necrosis factor-α inhibitors (TNF-i) was recorded, and the patients were considered current users if usage was noted in the patient record for half a year or longer before the evaluation period. Disease activity and functional status were assessed using the Bath Ankylosing Spondylitis Disease Activity Index (BASDAI) [17] and Bath Ankylosing Spondylitis Functional Index (BASFI) [18], respectively. Patient-reported outcomes were assessed using the Ankylosing Spondylitis Quality of Life Index (ASQOL) [19] and the short form-12 (SF-12) [20], which have physical component summary (PCS) and mental component summary (MCS) scores. ASQOL is a validated 18-item questionnaire that assesses the quality of life of AS patients. Comorbidities were evaluated using the rheumatic disease comorbidity index (RDCI) (range 0–10), representing the weighted sum score of common comorbidities (myocardial infarction, stroke, other heart diseases, lung disease, diabetes mellitus, hypertension, cancer, fracture, gastrointestinal ulcer, other gastrointestinal problems, and depression) [21].

Data on clinical characteristics were collected and evaluated independently by two rheumatologists (L.H.C. and M.S.L.), who had not participated in the study design, using a face-to-face questionnaire and medical records.

### Radiographic and laboratory data

The anteroposterior (AP) radiographs of the pelvis were obtained during outpatient visits, which were used to grade the damage of the sacroiliac joints (SIJs) according to the modified New York scoring method [16]. Radiographic damage of the spine was scored using the modified Stoke AS Spine Score (mSASSS) [22] from full-length radiographs of the spine. The bath ankylosing spondylitis radiology index of hip (BASRI-h) system was adopted to assess the severity of radiological involvement in the hip joint [23], which classifies the status of the hip joint into a 5-point scale from 0 to 4. The final score for radiographic damage of the SJI and hip was assessed on the side with more severe involvement. Laboratory data including human leukocyte antigen (HLA)-B27 status; serum erythrocyte sedimentation rate (ESR); and C-reactive protein (CRP), hemoglobin (HGB), and albumin (ALB) levels were also measured at enrollment.

### Adherence evaluations

The 18-item EAQ measures patients’ adherence to exercise therapy using a 4-point Likert scale (strongly disagree, 1 point; somewhat disagree, 2 points; somewhat agree, 3 points; strongly agree, 4 points). The final score was calculated by adding up all items, subtracting 18, and then dividing it by 54 in order to take it to a 0–1 scale; finally, it was multiplied by 100, obtaining a final range from 0 (no adherence) to 100 (perfect adherence) [8, 15].

### Statistical analysis

Descriptive data of categorical variables were expressed as percentages and frequencies, and of continuous variables, as mean and standard deviation (SD) or median and interquartile range ([IQR] 25–75%) if the data were skewed.

The correlations of EAQ score with clinical continuous variables and with ordinal variables were determined by correlation coefficient (*r*) in Pearson correlation analysis and Spearman rank correlation analysis, respectively. The cases in our series were grouped as good adherence and poor adherence using a cutoff score of 60, according to a previous study [7]. We conducted a univariate analysis to assess intergroup differences, using independent sample Student’s *t* tests or Mann–Whitney tests for continuous variables and chi-squared tests for dichotomous variables, respectively. All reported *p* values were two-tailed, with an alpha of 0.05. Next, a multivariate logistic regression model was used to assess the risk factors identified as significant in the analysis, and the odds ratio with 95% confidence interval (CI) and the associated *p* value were determined. In the multivariable binary logistic regression analysis, variables with a *p* value of ≤ 0.05 were assumed to be statistically significant determinants of poor adherence to exercise therapy. All analyses were performed using the IBM SPSS Statistics version 24.

## Results

A total of 185 outpatients completed the questionnaire. The mean EAQ score was 49.4 (IQR, 40.7–59.3); 146 patients (78.9%) were considered to have poor adherence (EAQ score ≤ 60), and 39 patients (21.1%) were considered to have good adherence (EAQ score >60). The patient sociodemographic, disease-related, radiographic, and laboratory parameters in the two groups are summarized in Table 1. The percentages of current NSAIDs, csDMARDs, and TNF-i use were significantly higher in the poor adherence group (*p*=0.001, *p*=0.027, *p*=0.018, respectively). The ESR and CRP levels were also significantly higher in the poor adherence group (*p*=0.025 and *p*=0.014, respectively). We found a statistically significant correlation between the EAQ score and age at outpatient visit (*r*=−0.203; *p*=0.005) and CRP (*r*=−0.250; *p*=0.001).

**Table 1:** Results of univariate statistical analysis between adherence groups

Parameters	All patients (*n*=185)	Good adherence group (*n*=39)	Poor adherence group (*n*=146)	*p* value
Sociodemographic parameters				
BMI	23.9±3.9	23.6±3.1	24.0±4.1	0.462
Sex				0.191
Male	153 (82.7%)	35 (89.7%)	118 (80.8%)	
Female	32 (17.3%)	4 (10.3%)	28 (19.2%)	
Age at outpatient (years)	29 (25, 33)	27 (23, 32)	29 (23, 34)	0.124
Family history, *n* (%)	58 (31.4%)	13 (33.3%)	45 (30.8%)	0.764
Currently employed, *n* (%)	137 (74.1%)	32 (82.1%)	105 (71.9%)	0.200
Education level				0.320
Primary school, *n* (%)	8 (4.3%)	0	8 (5.5%)	
Junior middle school, *n* (%)	44 (23.8%)	10 (25.6%)	34 (23.3%)	
Senior middle school or professional education, *n* (%)	51 (27.6%)	14 (35.9%)	37 (25.3%)	
University/graduate education, *n* (%)	82 (44.3%)	15 (38.5%)	67 (45.9%)	
Smoking habits, *n* (%)	100 (54.1%)	23 (59.0%)	77 (52.7%)	0.488
Disease-related parameters				
Age at onset (years)	22 (18, 25)	22 (18, 26)	23 (18, 25)	0.907
Disease duration (years)	7 (2, 12)	5 (3, 10)	7 (2, 13)	0.100
Diagnosis delay (years)	3 (0, 7)	3 (1, 7)	3 (0, 6)	0.712
VAS of global back pain	2.1 (0, 4.5)	2.0 (0, 4.5)	2.2 (0, 4.5)	0.903
EAMs				
Uveitis, *n* (%)	24 (13.0%)	3 (7.7%)	21 (14.4%)	0.269
IBD, *n* (%)	7 (3.8%)	0	7 (4.8%)	0.348
Psoriasis, *n* (%)	14 (7.6%)	1 (2.6%)	13 (8.9%)	0.307
Current medication use, *n* (%)	108 (58.4%)	13 (33.3%)	95 (65.1%)	<0.001
NSAIDs, *n* (%)	92 (49.7%)	10 (25.6%)	82 (56.2%)	0.001
csDMARDs, *n* (%)	55 (29.7%)	6 (15.4%)	49 (33.6%)	0.027
TNF-i, *n* (%)	46 (24.9%)	4 (10.3%)	42 (28.8%)	0.018
BASDAI	2.1 (1.0, 3.8)	2.1 (0.8, 3.9)	2.1 (1.0, 3.7)	0.741
BASFI	11 (2, 38)	5 (0, 37)	13 (3, 38)	0.083
SF-12 MCS	48.2 (42.0, 55.1)	50.2 (43.3, 55.1)	47.9 (41.5, 55.1)	0.477
SF-12 PCS	44.1 (34.8, 50.2)	44.2 (36.3, 52.4)	43.7 (34.2, 49.8)	0.206
ASQoL	3 (1, 8)	2 (1, 8)	4 (1, 8)	0.161
RDCI	0 (0, 2)	1 (0, 2)	0 (0, 2)	0.381
Laboratory parameters				
ESR (mm)	27 (23, 34)	24 (21, 33)	30 (23, 35)	0.025
CRP (mg/L)	24.5 (15.6, 34.5)	22.4 (10.4, 32.3)	24.5 (16.6, 35.4)	0.014
HGB (g/L)	135 (130, 143)	139 (134, 143)	134 (127, 142)	0.083
ALB (mg/L)	42.3 (41.2, 44.4)	42.4 (41.4, 44.5)	42.3 (40.9, 44.4)	0.285
HLA-B27 positive, *n* (%)	160 (86.5%)	35 (89.7%)	125 (85.6%)	0.503
Radiographic parameters				
mSASSS	33 (23, 41)	33 (23, 41)	33 (23, 41)	0.989
Sacroiliitis				0.441
Grade 1, *n* (%)	2 (1.1%)	0	2 (1.4%)	
Grade 2, *n* (%)	84 (45.4%)	19 (48.7%)	65 (44.5%)	
Grade 3, *n* (%)	80 (43.2%)	14 (35.9%)	66 (45.2%)	
Grade 4, *n* (%)	19 (10.3%)	6 (15.4%)	13 (8.9%)	
BASRI-h	2 (2, 3)	2 (2, 3)	2 (2, 3)	0.659

*BMI*, body mass index; *VAS*, visual analog scale; *EAMs*, extra-articular manifestations; *IBD*, inflammatory bowel disease; *NSAIDs*, nonsteroidal anti-inflammatory drugs; *csDMARDs*, conventional synthetic disease-modifying antirheumatic drugs; *TNF-i*, tumor necrosis factor-α inhibitor; *BASDAI*, Bath Ankylosing Spondylitis Disease Activity Index; *BASFI*, Bath Ankylosing Spondylitis Functional Index; *SF-12 MCS*, Short Form-12 Mental Component Summary; *SF-12 PCS*, Short Form-12 Physical Component Summary; *ASQoL*, Ankylosing Spondylitis Quality of Life; *RDCI*, Rheumatic Disease Comorbidity Index; *ESR*, erythrocyte sedimentation rate; *CRP*, C-reactive protein; *HGB*, hemoglobin; *ALB*, albumin; *HLA*, human leukocyte antigen; *mSASSS*, modified Stoke Ankylosing Spondylitis Spine Score; *BASRI-h*, Bath Ankylosing Spondylitis Radiology Index of Hip

The value of continuous variables was presented as mean ± standard deviation or median and quartile (25, 75%) and the categorical variables were based on presented as number plus percentage

The related factors identified as significant were introduced into the multivariate logistic regression model to assess the possible effects of these parameters on poor adherence to exercise therapy. The model revealed that only the current use of NSAIDs was a significantly associated factor (OR, 3.517; *p*=0.016; 95%CI, 1.259–9.827) (Table 2).

**Table 2:** The multivariate logistic regression model for poor adherence to physical exercises

Parameters	Multivariate analysis				
	*B*	Wald	*p*	Exp(B)	95%CI for Exp(B)
Sex	−0.584	0.774	0.379	0.558	0.152–2.049
Age at outpatient	0.028	0.451	0.502	1.029	0.947–1.118
Currently employed	−0.744	2.004	0.157	0.475	0.170–1.331
Disease duration	0.029	0.369	0.544	1.029	0.938–1.130
NSAIDs	1.258	5.757	0.016	3.517	1.259–9.827
csDMARDs	−0.293	0.203	0.652	0.746	0.209–2.663
TNF-i	0.984	2.317	0.128	2.675	0.754–9.492
BASFI	−0.002	0.038	0.845	0.998	0.981–1.016
SF-12 PCS	−0.014	0.261	0.609	0.986	0.935–1.040
ASQoL	0.021	0.117	0.733	1.021	0.906–1.151
ESR	0.010	0.240	0.624	1.010	0.972–1.049
CRP	0.024	1.373	0.241	1.024	0.984–1.067
HGB	−0.016	0.557	0.455	0.984	0.944–1.026

*NSAIDs*, nonsteroidal anti-inflammatory drugs; *csDMARDs*, conventional synthetic disease-modifying antirheumatic drugs; *TNF-i*, tumor necrosis factor-α inhibitor; *BASFI*, Bath Ankylosing Spondylitis Functional Index; *SF-12 PCS*, Short Form-12 Physical Component Summary; *ASQoL*, Ankylosing Spondylitis Quality of Life; *ESR*, Erythrocyte Sedimentation Rate; *CRP*, C-reactive protein; *HGB*, hemoglobin

## Discussion

Exercise therapy is a cornerstone of current global treatment strategies for patients with AS. The primary goals of exercise therapy for patients with AS are to control pain, improve the mobility and strength of the involved joints, prevent or decrease deformity of the axial skeleton and peripheral joints, and ultimately improve the overall function and quality of life [24, 25]. Our research was the first cross-sectional study to assess adherence to exercise therapy of outpatients with AS and analyze its relationship with sociodemographic, disease-related, radiographic, and laboratory parameters.

There is no gold standard for the evaluation of adherence. Clinically, adherence to exercise therapy is measured using various methods, making direct evaluation difficult [26]. In a study by Sang et al. [14], the criterion they selected as adherence to the standard exercise therapy was to exercise at least 30 min per day and perform back exercise at least 5 days per week. Obviously, this was only a qualitative evaluation criterion. The EAQ is an easy-to-understand, quick, and self-administered questionnaire; its reliability and validity in clinical settings have been confirmed in previous studies [8, 15]. Consequently, we used EAQ as our assessment tool for adherence to exercise therapy in patients with AS.

The percentage of adherence to exercise therapy varied widely from 51 to 95% in published literature [15, 26]. Arturi et al. [8] investigated the adherence of exercise among 59 patients with AS using the EAQ. The median value of EAQ was 40.7 (IQR, 5.6–77.8). Using a cutoff EAQ value higher than 60, they dichotomized the patients as adherent and non-adherent, and 53 patients adhered to the exercise regimen. In contrast, the percentage of adherence to exercise therapy in our series was only 79% (146/185), well below the level mentioned above.

Medication adherence of patients with AS has been evaluated in previous studies, and the associated factors include advanced age, quality of life, patients’ beliefs about medicines and illness perceptions, choice of drugs, and route of administration [6–9, 27]. Unfortunately, no study has specifically addressed the determinant factors for adherence to exercise therapy. In our series, patient sociodemographic, disease-related, radiographic, and laboratory parameters were identified. We found a weak correlation between EAQ score and age at outpatient visits (*r*=−0.203; *p*=0.005), but the latter was not statistically significantly different between the good and poor adherence group. The level of CRP was negatively correlated with the EAQ score (*r*=−0.250; *p*=0.001), and the intergroup difference was also statistically significant (*p*=0.014). Unfortunately, we did not find any statistical significance for CRP (*p*=0.241) in our multivariate logistic regression model. Measurement of CRP level is one of the most widely used methods to assess disease activity of AS and was confirmed an independent risk factor for axial physical mobility and radiographic progression in patients with AS [28, 29]. AS patients with elevated CRP levels present poor physical mobility and new bone formation of spine, which may have an adverse effect on the adherence to exercise therapy.

In our study, the current use of NSAIDs was identified as the statistically significant factor for poor adherence to exercise therapy. This was an interesting finding, and our explanations are as follows. NSAIDs have always been considered the first-line therapy for patients with AS and can be highly effective for treating axial and peripheral symptoms, including pain, stiffness, and limited range of motion. Moreover, a series of studies provided supportive evidence that NSAIDs may slow the progression of bony changes in the spine in AS [30, 31]. Consequently, patients with AS tend to have better symptom control after receiving NSAIDs, including relief of pain and morning stiffness, and improvement of range of motion, which negatively impacts adherence to exercise therapy. Similarly, we also noted significant differences in the current use of csDMARDs and TNF-i between the good adherence and poor adherence groups, although they were not significant determinant factors in our multivariate logistic regression model.

The main limitation of our study was its single-center cross-sectional nature. In fact, adherence is a dynamic process that tends to decline over time. The results of the current study may not be generalizable to the population at large or to other regions, as our patients were enrolled from a single center. Second, the sample size was too small to find significant correlations with other risk factors. Third, it was impossible to assess all risk factors for poor adherence to physical exercise, including some sociodemographic factors, such as marital status, income level, and dietary pattern, and disease-related parameter, such as the Bath Ankylosing Spondylitis Metrology Index (BASMI).

In conclusion, lack of adherence to exercise therapy is a major concern in the treatment of patients with AS. According to our study, outpatients with AS have an unacceptable level of adherence to exercise therapy, and current use of NSAIDs was identified as the only significant associated factor with poor adherence. Clinically, there is an urgent need to develop comprehensive strategies to improve adherence to exercise therapy in patients with AS, and future studies can aim to determine the reasons behind the lack of adherence to exercise therapy.

## Data Availability

The datasets used and/or analyzed during the current study are available from the corresponding author on reasonable request.
